# Epigenetic Liquid Biopsy Enables Universal Mutation-Agnostic Molecular Surveillance for High-Risk Neuroblastoma

**DOI:** 10.1158/1078-0432.CCR-26-0457

**Published:** 2026-07-01

**Authors:** Nurit Gal-Mark, Assaf Grunwald, Valid Gahramanov, Michal Hameiri-Grossman, Elena Shinderman-Maman, Dafna Gaas, Keren Shichrur, Jacques Mordoukh, Nino Oniashvili, Eva Chausky Barzakh, Aviv Sever, Shirah Amar, Shifra Ash, Yehudit Birger, Shai Izraeli, Yuval Ebenstein, Esther R. Berko

**Affiliations:** 1 The Rina Zaizov Division of Pediatric Hematology-Oncology, Schneider Children’s Medical Center, Petach Tikva, Israel.; 2 Felsenstein Medical Research Center, Tel Aviv University, Rabin Medical Center, Petah Tikva, Israel.; 3 Department of Physical Chemistry, School of Chemistry, Tel Aviv University, Tel Aviv, Israel.; 4 Department of Human Genetics and Computational Medicine, Gray Faculty of Medical and Health Sciences, Tel Aviv University, Tel Aviv, Israel.; 5 Dr. Miriam and Sheldon G. Adelson School of Medicine, Ariel University, Ariel, Israel.; 6 Division of Oncology, Center for Childhood Cancer Research, Children’s Hospital of Philadelphia, Philadelphia, Pennsylvania.

## Abstract

**Purpose:**

Liquid biopsy monitoring in pediatric solid tumors is limited by low mutational burden and lack of trackable genomic drivers. We sought to develop a mutation-agnostic, methylation-based liquid biopsy framework enabling universal molecular surveillance of high-risk neuroblastoma.

**Experimental Design:**

Using whole-genome Oxford Nanopore Technologies sequencing of high-risk neuroblastoma tumors, we compared tumor-derived methylation profiles with a comprehensive atlas of normal human cell types and identified 72 neuroblastoma-specific differentially methylated regions (meNBL) that were reliably detectable in cell-free DNA (cfDNA). Marker robustness and specificity were validated using independent neuroblastoma methylation datasets and assessed against methylation profiles from other cancer types. We established neuroblastoma as a distinct methylation entity within the reference atlas by integrating a panel of 25 meNBLs, enabling quantitative estimation of tumor-derived cfDNA. Assay performance was evaluated across diagnostic, remission, relapse, and healthy control samples and compared with mutation-based and copy number–based approaches.

**Results:**

Neuroblastoma-derived cfDNA was consistently detected at diagnosis and relapse but was absent in healthy controls and during confirmed remission. Methylation-based deconvolution demonstrated high specificity, with no detectable background signal in controls, and improved performance relative to copy number–based tumor fraction estimation. Longitudinal profiling enabled early molecular detection of relapse and reliable disease monitoring.

**Conclusions:**

We establish a robust, mutation-independent methylation-based liquid biopsy strategy for neuroblastoma that enables accurate, quantitative disease monitoring across all high-risk patients, including those lacking trackable genomic alterations. This approach supports the clinical translation of methylation-based cfDNA deconvolution as a broadly applicable platform for pediatric precision oncology.

**Translational Relevance**

The majority of pediatric solid tumors lack trackable genetic alterations, limiting the utility of current liquid biopsy approaches for disease monitoring. This study establishes a mutation-agnostic, methylation-based liquid biopsy framework that enables sensitive and quantitative detection of neuroblastoma-derived circulating tumor DNA (ctDNA) across all high-risk patients. By integrating tumor-specific methylation signatures into a reference atlas and applying nanopore sequencing, this approach allows for accurate disease burden assessment, early relapse detection, and longitudinal monitoring without reliance on trackable genetic drivers. Importantly, this platform outperforms conventional copy number–based methods and demonstrates high specificity with no detectable background signal in healthy controls. These findings support the clinical translation of methylation-based cell-free DNA monitoring as a broadly applicable, minimally invasive tool that may enable earlier therapeutic intervention, reduce dependence on imaging and sedation-intensive procedures, and facilitate real-time precision management of children with high-risk neuroblastoma.

## Introduction

Neuroblastoma, a malignancy of the developing sympathetic nervous system, is the most common extracranial solid tumor of early childhood. The disease is highly heterogeneous, with clinical behavior ranging from spontaneous regression to aggressive therapy-resistant progression (1). Despite extensive and highly morbid treatments, event-free survival in high-risk neuroblastoma remains ∼50%, and outcomes following relapse are dismal.

Like other pediatric solid tumors, high-risk neuroblastoma is characterized by a relative paucity of pathogenic single-nucleotide variants (SNV) at diagnosis (2). Although several genomic alterations associated with aggressive disease have been described, most are not amenable to standardized SNV-based molecular tracking. *MYCN* amplification is a well-established driver of high-risk neuroblastoma and confers aggressive biology. Activating aberrations in the anaplastic lymphoma kinase (*ALK*) gene occur in ∼20% of patients, portend inferior prognosis, and remain the only currently tractable therapeutic target in this disease (3). Additional alterations involving the *TERT* promoter locus, *ATRX*, and the RAS–MAPK pathway occur in subsets of tumors and are associated with inferior outcomes (4). However, the majority of high-risk neuroblastoma tumors lack recurrent, clonal SNVs suitable for longitudinal molecular monitoring and are instead characterized only by classic segmental chromosomal aberrations (SCA; ref. 5).

Liquid biopsy analysis of ctDNA has emerged as a powerful, minimally invasive approach to capture tumor genetic heterogeneity, monitor disease burden, and detect clonal evolution in high-risk neuroblastoma (6, 7). Although ultradeep targeted sequencing panels and mutation-specific PCR assays have demonstrated clinical utility (6–8), their application is limited to approximately 50% of patients whose tumors harbor trackable driver oncogenic alterations. This limitation underscores the unmet need for universal mutation-independent biomarkers for liquid biopsy-based monitoring across the full high-risk neuroblastoma population.

DNA methylation patterns are highly cell-type specific, stable across cell divisions, and universally present, making them compelling candidates for mutation-agnostic liquid biopsy approaches. Prior methylation studies in neuroblastoma have largely focused on identifying prognostic signatures by comparing high-risk tumors with low-risk disease or normal tissues (9–12). While informative, these approaches were not designed to enable sensitive and specific detection of tumor-derived DNA within the complex mixture of cell-free DNA (cfDNA) and typically interrogate only a subset of genomic loci. Here, we adopt an alternative strategy. Building on methylation-based deconvolution frameworks established by Dor and colleagues (13, 14), we identify differentially methylated regions (DMR) that uniquely distinguish neuroblastoma from all other normal human cell types represented in the comprehensive methylation atlas. Using Oxford Nanopore Technologies (ONT) sequencing, which enables direct methylome profiling from native DNA while simultaneously capturing SNVs and copy-number alterations (CNA), we integrate high-risk neuroblastoma as a distinct entity within the reference atlas. We show that this approach enables the sensitive and quantitative detection of neuroblastoma-derived cfDNA across different disease states, supporting methylation-based deconvolution as a robust and universal framework for longitudinal monitoring of neuroblastoma.

## Materials and Methods

### Patient cohort and samples

In this single-site study, pediatric patient samples were obtained from individuals treated at Schneider Children’s Medical Center of Israel (2016–2025) in accordance with the Declaration of Helsinki, local guidelines, and Rabin Medical Center Institutional Review Board approval #4370-RMC, with written informed consent (#0012-08-RMC). Our localized cohort subjected to ONT sequencing included 7 high-risk neuroblastoma tumor samples, 11 cfDNA samples collected at diagnosis, and 17 longitudinal cfDNA samples obtained from 7 patients encompassing both *MYCN*-amplified and nonamplified subtypes. Reference samples included cfDNA from eight healthy donors (five adult and three pediatric) and three peripheral blood lymphocyte (PBL) pools, each derived from the genomic DNA (gDNA) of 10 individuals (Supplementary Tables S1 and S2).

### DNA isolation

gDNA was extracted from fresh-frozen (FF) tumor tissue using spin columns (QIAamp DNA Micro Kit, QIAGEN) according to the manufacturer’s instructions. Tumor content was verified either by tumor fraction estimates derived from ichorCNA (RRID:SCR_024768) or by the observed variant allele frequency (VAF) in cases where a tumor-specific mutation was available. All samples indicated a tumor fraction of at least 40%. The quantification of the isolated DNA was conducted using a Qubit Flex fluorometer (RRID:SCR_020553) with a 1× double-stranded DNA (dsDNA) HS Assay Kit (Invitrogen, Thermo Fisher Scientific). Whole blood was collected in EDTA Vacutainer tubes (BD Biosciences) and separated on the same day. Separation of plasma was performed by centrifugation for 10 minutes at 1,600 *g* at room temperature, with the brake set to “off,” followed by a subsequent centrifugation step of 10 minutes at 1,600 *g* at room temperature. The plasma was stored at −80°C until it was processed for cfDNA extraction. Isolation of cfDNA was performed starting from 400 μL to 3.5 mL of plasma. Smaller sample volumes were adjusted to 1 mL by adding 1× PBS (Sartorius). Beginning in April 2024, for patients with neuroblastoma, blood was collected in PAXgene Blood ccfDNA Tubes (QIAGEN); plasma from three patients with neuroblastoma was processed using the same protocol as for EDTA samples, except that the tubes allowed for longer storage before plasma separation.

After plasma was thawed and centrifuged for 10 minutes at 20,000 *g* at 4°C, cfDNA was extracted using the QIAamp Circulating Nucleic Acid Kit (QIAGEN) with the Qiavac 24 system, according to the manufacturer’s recommendations. cfDNA concentration was measured using the Qubit Flex fluorometer with a 1× dsDNA HS Assay Kit (Invitrogen, Thermo Fisher Scientific, RRID:SCR_020553). If cfDNA concentration was low, but sufficient volume was available, samples were concentrated up to twofold using SpeedVac vacuum centrifugation. Size distribution of the cfDNA fragments was measured on a 4200 TapeStation system (Agilent Technologies).

### Library preparation and nanopore sequencing

Library preparation was performed using the Ligation Sequencing Kit V14 (SQK-LSK114, ONT) or the Native Barcoding Kit 24 V14 (SQK-NBD114.24, ONT) following the manufacturer’s instructions, with modifications implemented as needed to accommodate limited input material. Libraries were successfully generated from as little as 1.5 μg of gDNA and 8 ng of cfDNA. Most samples were run on single flow cells to maximize sequencing yield (Supplementary Tables S1–S3). For one cfDNA sample, an overnight ligation step was tested in parallel. However, as this modification did not yield notable differences in sequencing performance, it was not applied in subsequent library preparations. Sequencing was performed on a P2Solo device using R10.4.1 Flow Cells (FLO-MIN114, ONT).

### Base calling and alignment

Base calling and alignment were performed using ONT MinKNOW (version 24.06.16, ONT), with the “modified base calling” option enabled. The human reference genome hg38 was used for alignment. Reads from individual aligned BAM files were merged, sorted, and indexed using samtools (RRID:SCR_002105 version 1.10). As the reference atlas, used in downstream analysis, is based on bisulfite sequencing [which cannot distinguish 5-methylcytosine (5mC) from 5-hydroxymethylcytosine (5hmC)], our nanopore-detected 5hmC signals were converted to 5mC-equivalent using Modkit’s adjust-mods function (RRID:SCR_028163).

### Genetic analysis

SNVs and structural variants (SV) were identified from whole-genome sequencing data across 60 genes selected through a comprehensive review of published genomic datasets on high-risk neuroblastoma, as detailed in Supplementary Table S4. Variant calling was performed using Clair3 (RRID:SCR_026063) and Sniffles2 (RRID:SCR_017619) via the EPI2ME wf-human-variation workflow. Single-nucleotide polymorphisms (SNP) were subsequently annotated using ClinVar (RRID:SCR_006169). Given the limited availability of comprehensive SV annotation databases for ONT data, a healthy control SV reference was constructed using PBL samples. SVs were called in the PBL samples using the same pipeline, merged using bcftools (RRID:SCR_005227, version 1.3.1), and treated as a nonpathogenic reference set. Only SVs unique to the tumor samples (i.e., not overlapping with the control SVs) were retained for downstream interpretation. Detection of clinically relevant mutations in these samples was supported by targeted oncogene panels and/or Droplet Digital PCR (ddPCR), in addition to the genomic sequencing data. Copy-number variations (CNV) were detected using ichorCNA, applying 500 kbp bin sizes and default settings, including the package provided panel of normals (PoN; ref. 15). To assess the impact of PoN selection, ichorCNA analyses were additionally performed using an ONT-derived PoN constructed from 12 healthy control samples and in a PoN-free configuration. SCAs were annotated as per clinical criteria. ONT sequencing demonstrated concordance with available clinical assessments, detecting the same genetic aberrations previously identified by SNP array or targeted panel (Oncomine) sequencing, when available, with two mutations observed below standard thresholds (Supplementary Table S1).

### DNA methylation analysis

DNA methylation β values, representing methylation levels (0 = unmethylated to 1 = fully methylated), were calculated per CpG site using Modkit (RRID:SCR_028163, version: 0.1.12, ONT), with the –combine-strands and –cpg flags. Data analysis, including principal component analysis (PCA), hierarchical clustering, correlation heatmaps, and violin plots, was performed in Python 3.10.7 using the pandas (RRID:SCR_018214), scikit-learn (RRID:SCR_002577), matplotlib (RRID:SCR_008624), and seaborn (RRID:SCR_018132) libraries.

### Neuroblastoma-specific marker identification and selection

Marker identification was based on the framework and reference data described by Loyfer and colleagues (13). Briefly, BAM files were converted to “beta” and “pat” formats using wgbstools with the bam2pat option and the –nanopore flag (13). The resulting PAT files (one per tumor DNA sample) were used in conjunction with a tissue-specific methylation dataset (GSE186458), which comprises 206 samples from 36 available different tissues. To identify uniformly methylated blocks, we applied the wgbstools segment option with parameters –min_cpg 3 –max_bp 10000, resulting in ∼4.3M blocks with a mean of 180 bp and a median of 379 bp. This script detects genomic segments that exhibit consistent CpG methylation levels across all samples, including both our neuroblastoma tumors and the external reference dataset.

Differential methylation analysis was performed using the wgbstools find markers command with default parameters. Neuroblastoma samples were compared against all other tissues in the methylation dataset. Regions were considered significant if they met a nominal *P* value threshold of 0.05 and the Δβ value (difference between neuroblastoma methylation and methylation in all other tissues) was in the top third percentile of the whole-genome Δβ distribution. In the study by Loyfer and colleagues, 3 to 4 replicates per cell type were sufficient to infer robust methylation patterns for biomarker identification. The inclusion of seven neuroblastoma tumors in our study aligns with their methodology and provides adequate representation. Given that neuroblastoma arises from neural crest cells, we repeated the analysis excluding neuronal cell types from the reference atlas, identifying additional DMRs associated with neuronal origin. Most regions were shared between the analyses including and excluding neuronal tissues (Supplementary Fig. S1). Regions overlapping centromeres were excluded using bedtools (RRID:SCR_006646) to intersect with GRCh38 centromere annotations (GRCh38.GCA_000001405.2_centromere_acen).

From an initial set of 188 DMRs, we first selected 76 regions exhibiting low mean methylation in healthy cfDNA (<10%). We then excluded two DMRs with high methylation in PBL pools (>20%), as gDNA contamination in cfDNA can elevate overall methylation levels. Two additional DMRs were manually excluded: one lacking sufficient coverage in ≥5/12 neuroblastoma cfDNA samples and the other featuring a small CpG cluster (2 CpGs within a locus <80 bp). This analysis yielded a final set of 72 DMRs termed meNBLs. The methylation score was calculated using bedtools “map” with the -c mean flag to obtain an average value for all the different CpGs in the marker region. This was also done for public data obtained from the TARGET project (16).

The Genomic Regions Enrichment of Annotations Tool (RRID:SCR_005807) was used to affiliate markers to genes based on regulatory domains and genomic proximity, using default parameters. Gene Ontology (GO) enrichment analysis (RRID:SCR_002811) was carried out on the filtered subset of our DMRs (Supplementary Fig. S2). To enable comparison with methylation profiles from an independent ONT sequencing dataset of advanced cancers, only tumors from patients younger than 30 years were included to better approximate the age distribution of our pediatric cohort (17). We further supplemented this with adult breast and lung cancer samples (Supplementary Table S5).

### GO enrichment analysis

GO enrichment analysis (RRID:SCR_002811) was performed in R using the clusterProfiler package (RRID:SCR_016884) with default parameters. Enrichment significance was determined using a hypergeometric test with Benjamini–Hochberg correction for multiple testing. GO terms with adjusted *P* values (FDR) < 0.05 were considered significant.

### Deconvolution

Twenty five selected meNBLs were added to the existing atlas (13) using uxmtools “build” option with the –use_um and –rlen 4 options to generate a modified atlas including the neuroblastoma entity. Cell of origin deconvolution was performed using the uxmtools deconv option with our novel neuroblastoma atlas.

To ensure balanced representation of the different tissues in the modified atlas, which originally contained 25 markers per tissue type, we selected a subset of 25 markers from the 72 meNBL markers, prioritized based on the criteria described below to ensure biological relevance and robust signal characteristics. The selection maintained proportions between neuronal and nonneuronal features, mirroring the initial identification of 188 DMRs. For nonneuronal markers, selection was based on a multicriteria ranking approach. Candidate DMRs were evaluated across five criteria: (i) highest CpG cluster span indicative of biologically meaningful regulatory regions, (ii) lowest mean methylation in healthy control cfDNA, (iii) lowest mean methylation in other advanced cancers, (iv) highest mean methylation difference between tumor and healthy cfDNA, and (v) promoter-proximal location (±5 kb). Top-ranking candidates from the upper quartile of each criterion, with preference given to DMRs satisfying multiple criteria and demonstrating robust and consistent signal characteristics based on manual review, were selected for inclusion. Neuronal markers were prioritized based on brain tissue differential expression (GTEx database, RRID:SCR_001618) and promoter location, ensuring a heterogeneous and balanced panel.

### ddPCR

The QX200 ddPCR System (RRID:SCR_019707, Bio-Rad Laboratories) was used to determine genomic aberrations in neuroblastoma tissue or cfDNA. The *ALK* R1275Q (exon 25, position 3,824, G > A) mutation was detected using Bio-Rad wet-lab assays dHsaCP250606768 and dHsaCP250606769. Analysis of the copy number statuses of *MYCN* (2p24.3) or *ALK* (2p23.2 to 2p23.1) was performed with two reference genes: *NAGK* (2p13.3) and *AFF3* (2q11.2), in triplex ddPCR assays as described (8). PCRs were performed on a C1000 thermocycler (Bio-Rad Laboratories), with an annealing temperature of 55°C for the *ALK* R1275Q mutation detection assay and 58°C for the *MYCN*/*ALK* copy-number assays as described (8). All ddPCR assays contained appropriate nontemplate, positive and negative controls in each run. Droplet reactions were assessed in the QX200 ddPCR Droplet Reader. Assays were analyzed using QX Manager Software version 2.2 (Bio-Rad Laboratories).

### Neuroblastoma panel sequencing

Tumor profiling was performed using the pan-pediatric cancer panel, Oncomine Childhood Cancer Research Assay (Ion Torrent, Thermo Fisher Scientific), according to the manufacturer’s protocol. This tool analyzes the mutational status of 200 genes, including 82 mutation hotspots, 24 CNV targets, 44 genes with full exome coverage, and an RNA fusion panel of 97 genes. DNA and RNA libraries were generated using Ion AmpliSeq Library Preparation on the Ion Chef System (Thermo Fisher Scientific, RRID:SCR_026507). Complementary DNA synthesis prior to library preparation for the RNA panel was carried out using SuperScript VILO Reverse Transcriptase (Thermo Fisher Scientific). Sequencing was performed on the Ion Torrent S5 (Thermo Fisher Scientific, RRID:SCR_027446). Variants were identified and annotated by the Thermo Fisher Ion Reporter. A variant was accepted for further analysis if its coverage was at least 500 reads and the VAF was greater than 5% (or 2% for hotspots). Amplification was reported when CN was above 8 at the 5% confidence level. Fusion genes were recognized as such if they had at least 10,000 per million reads. The identified variants were evaluated by different databases to analyze their clinical significance.

### SNP array

Array analysis was performed using the CytoScan HD array (Affymetrix) according to the manufacturer’s recommendations (Affymetrix manual protocol Affymetrix Cytogenetics Copy Number Assay P/N 703038 Rev. 3). The raw data were processed using Chromosome Analysis Suite 4.5.0.34.

## Results

### Identification of novel neuroblastoma-specific epigenetic markers

**Figure 1.**
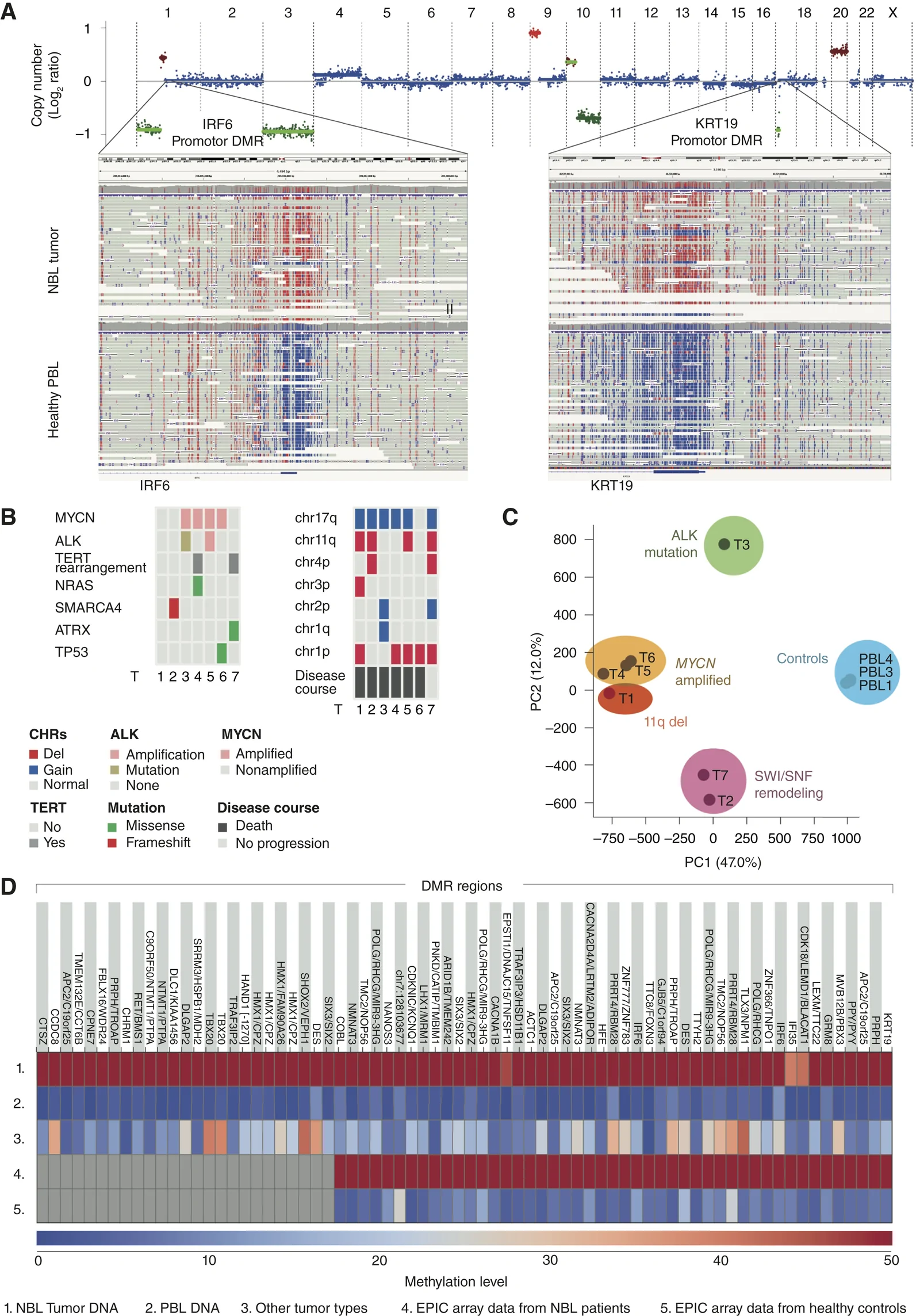
Integrated genetic and epigenetic profiling of neuroblastoma using whole-genome ONT sequencing. **A,** Whole-genome ONT sequencing of tumor DNA provides simultaneous genetic and epigenetic information, illustrated here for patient T6. The data enable detection of large-scale genetic alterations, shown as a CNV profile at the top, and identification of DMRs relative to control samples (bottom). Bottom, Two representative promoter-associated DMRs: the newly identified IRF6 promoter on chromosome 1 and the previously reported KRT19 promoter on chromosome 17. Individual native DNA molecules aligned to each locus are displayed, with CpG sites colored red (methylated) or blue (unmethylated). **B,** Oncoplot: Major genetic alterations associated with neuroblastoma were identified by whole-genome ONT sequencing and summarized across the seven sequenced tumors. The oncoplot displays clinically relevant genomic events, including gene amplifications, mutations, deletions, chromosomal copy-number aberrations, and clinical disease course annotations. **C,** PCA of genome-wide DNA methylation profiles from the seven neuroblastoma tumors reveals clustering according to key genomic alterations, including MYCN amplification and 11q deletion, ALK mutation, and SWI/SNF remodeling, with control samples forming a distinct cluster. **D,** Neuroblastoma DMR heatmap: DNA methylation data from the seven neuroblastoma tumors were compared against a reference atlas comprising methylomes from 36 healthy tissue types to identify pan-neuroblastoma–specific DMRs. Methylation levels across the 72 identified DMRs are shown in the heatmap, with colors indicating average methylation levels (red, high methylation; blue, low methylation); values represent group-level averages for each column, arranged in rows representing from top to bottom: the seven sequenced neuroblastoma tumors, PBL samples as healthy controls, public methylation data from other advanced tumor types, and previously published neuroblastoma and healthy control methylomes generated using Illumina EPIC arrays. Neuroblastoma DMRs not interrogated by the EPIC array are shown in gray. NBL, neuroblastoma.

To identify robust neuroblastoma CpG DMRs, we performed ONT sequencing on seven high-risk neuroblastoma tumor samples, achieving a median coverage of 32× genome-wide. The seven tumor samples were selected to represent the major molecular subtypes and genetic alterations characteristic of high-risk neuroblastoma (Supplementary Table S1). ONT sequencing profiled concurrent genome-wide genetic (SNV and CNV) and epigenetic (CpG methylation) data from each sample (Fig. 1A). All known clinically relevant SNVs and SCAs previously identified by clinical testing with multiple orthogonal diagnostic approaches were detectable in the ONT sequencing data (Fig. 1B). Additionally, the long-read ONT approach enabled the identification of novel SVs not seen in clinical testing panels (Supplementary Table S1). Genome-wide DNA methylation profiling revealed globally reduced levels of DNA methylation in neuroblastoma tumor gDNA compared with control mixed PBL DNA samples (Supplementary Fig. S3A), consistent with the well-established global hypomethylation observed across many cancer types (18). Hierarchical clustering and PCA based on global methylation patterns clearly distinguished neuroblastoma tumors from control samples and further subdivided neuroblastoma tumors according to molecular subtypes, in agreement with previously reported classifications (Fig. 1C; Supplementary Fig. S3B; ref. 19).

Leveraging the methylation atlas of normal human cell types (13), we identified 72 novel neuroblastoma-specific DMRs, herein referred to as meNBLs (Fig. 1D; Supplementary Fig. S4; Supplementary Table S6; see “Patients and Methods” for DMR selection criteria). GO analysis revealed that genes associated with neuroblastoma-specific DMRs were significantly enriched in biological processes related to embryonic organ development, cognition, and neuronal maturation, consistent with the expected lineage and developmental origin of neuroblastoma (Supplementary Fig. S2; Supplementary Table S7). The 72 meNBLs identified were found to be distributed across various genomic regions, including promoters, gene bodies, 3′ untranslated regions, and intergenic regions, with several mapping to long noncoding RNAs (Supplementary Fig. S5). In addition to established DNA methylation biomarkers in neuroblastoma, such as *PRPH* and *KRT19* (11), several novel candidates were identified. These include transcription factors such as *HAND1*, *SIX3*, *SHOX2*, *HMX1*, *IRF6*, and *TBX20*, many of which are known to play roles in embryonic and neuronal development. Additionally, genes encoding calcium and potassium channels, *CACNA1B* and *CACNA2D4*, were also identified, all of which are involved in neuronal signaling. The involvement of these developmental regulators aligns with the embryonic origin of neuroblastoma and highlights pathways in which dysregulation may contribute to aberrant differentiation and tumorigenesis. Notably, although we observed global hypomethylation in neuroblastoma tumor genomes, the neuroblastoma-specific DMRs were predominantly hypermethylated relative to other tissues in the human methylation atlas (Supplementary Table S8). The existence of highly methylated regions within a globally hypomethylated background has likewise been reported in previous studies of neuroblastoma (17, 20).

To further evaluate the robustness of our meNBL panel, we used publicly available EPIC array data from the TARGET neuroblastoma cohort (a clinically annotated cohort enriched for high-risk disease) comprising 205 neuroblastoma tumors and 152 healthy controls (16). Many of the EPIC array features overlap with our meNBL panel and display similar distinct methylation-level differences between neuroblastoma and controls (Fig. 1D). Notably, ∼30% of our meNBLs (highlighted in gray in Fig. 1D) are not covered by the EPIC array, highlighting the potential of our approach in discovering novel diagnostic biomarkers and potential therapeutic targets that are inaccessible to EPIC arrays. We compared the meNBLs’ methylation levels with a published long-read sequencing cohort of 189 patient tumors spanning multiple cancer types, generated using the ONT platform, which provides a broadly comparable advanced cancer reference set (17). To better match the age profile of our pediatric tumors, we selected samples from patients below the age of 30, as well as breast and lung cancer samples (Supplementary Table S5). This benchmarking analysis confirmed that our selected meNBLs contain both DMRs that are (i) unique to neuroblastoma, reflecting the neuronal cell of origin of neuroblastoma when compared with other malignancies, and (ii) shared DMRs with other cancer types. The combination of these markers enables accurate detection of this peripheral nerve malignancy by confirming both its oncogenic nature and its neuronal origin (Fig. 1D).

### Application of meNBLs to liquid biopsy–based disease detection

**Figure 2.**
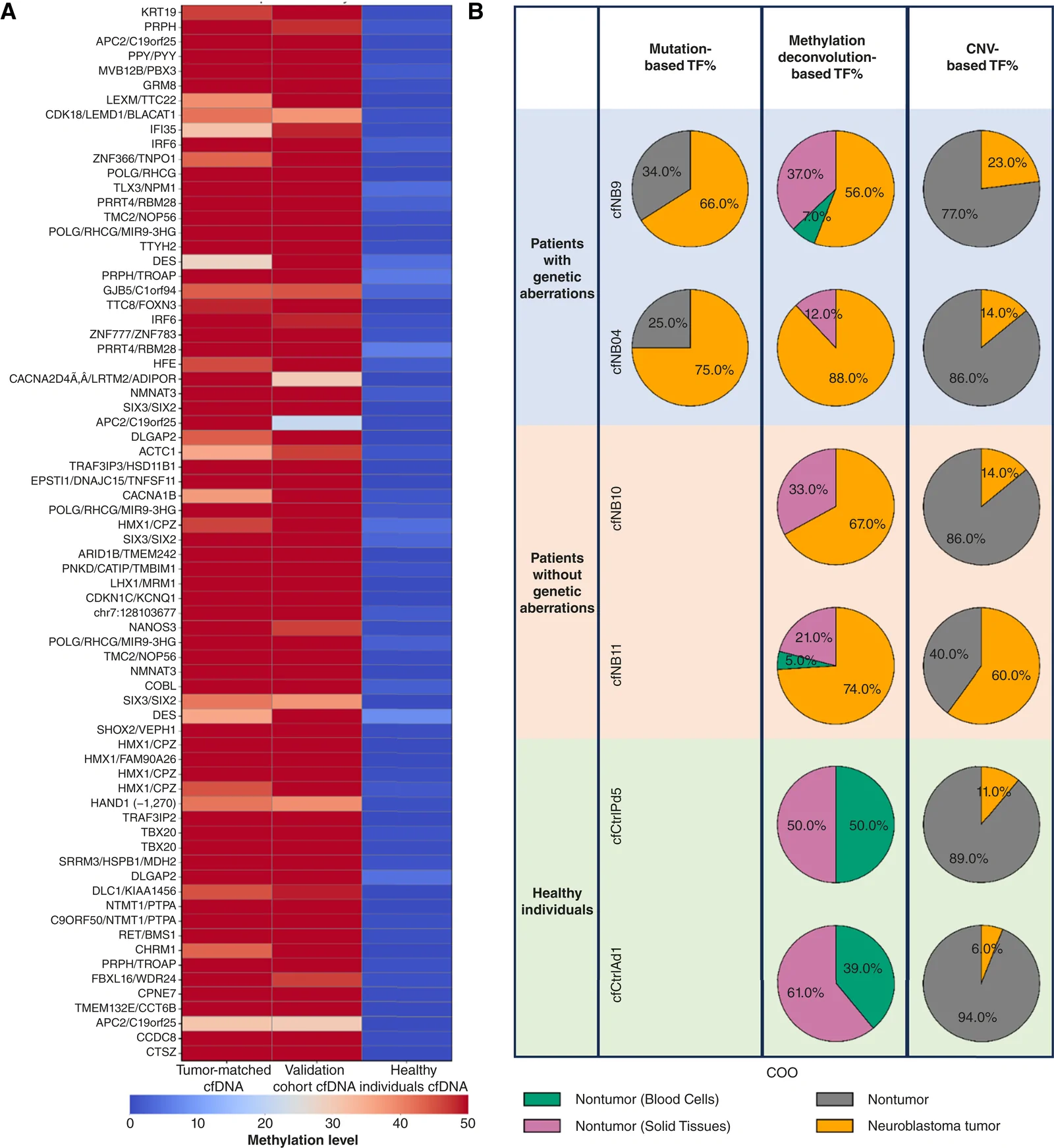
Detection of neuroblastoma markers in cfDNA samples. **A,** Heatmap showing average DNA methylation levels across the 72 identified DMRs. Rows correspond to individual meNBL markers, and columns represent cfDNA sample groups: matched cfDNA from patients with sequenced tumor tissue, cfDNA from an independent validation cohort of patients with neuroblastoma, and cfDNA from healthy individuals. Colors indicate mean methylation levels (red, high methylation; blue, low methylation). Values represent group-level averages across samples within each category. **B,** Tumor fraction (TF%) estimates in cfDNA for representative individuals, stratified by clinical and genomic status, using three independent approaches. Columns show TF% estimation based on (left) somatic mutation allele frequencies, applicable only to patients with identifiable tumor mutations; (middle) methylation-based deconvolution using our novel meNBL markers; and (right) CNV-based estimation using ichorCNA. Pie charts depict the relative contribution of tumor-derived cfDNA (orange-red) and nontumor cfDNA fractions. Only the methylation-based approach further resolves the nontumor fraction into cell of origin components.

To assess whether meNBLs can be detected in cfDNA and utilized for liquid biopsy, we performed ONT sequencing on cfDNA from five samples matched to sequenced tumor tissues and on an additional seven cfDNA samples collected at diagnosis, which served as an independent validation cohort. Additionally, we sequenced cfDNA from eight healthy individuals to serve as controls (median coverage of 2.8×, Supplementary Table S2). Importantly, analysis of meNBL DMRs showed concordance across all loci in all high-risk patients with neuroblastoma, with no statistically significant differences in methylation levels observed between the matched and independent cohorts (*t* test, *P* value threshold 0.05; Fig. 2A). These findings confirm that the selected markers are not restricted to the initial patients whose tumors were used for marker discovery, supporting their broader applicability as neuroblastoma-specific biomarkers.

To develop a quantitative neuroblastoma cfDNA assay, we leveraged a cell-of-origin deconvolution approach (13, 14). This method enables quantifying the contribution of different cell types to the total cfDNA content extracted from a given sample. We hypothesized that by defining neuroblastoma as an additional cell type in the human methylation atlas, we could detect neuroblastoma signatures that correlate with the existence and state of the disease. As the atlas includes 25 markers per cell type, we selected 25 meNBLs from the 72 neuroblastoma-specific markers and incorporated them into the atlas as a distinct neuroblastoma entity (see “Patients and Methods,” Supplementary Table S9). We performed methylation-based deconvolution on all our cfDNA samples using our modified atlas. No neuroblastoma fraction was detected for the control cfDNA samples, whereas varying amounts of neuroblastoma ctDNA were detected for all of the patient liquid biopsies (Fig. 2B; Supplementary Table S10). To validate the reliability of our tumor fraction quantification (TF%), we compared neuroblastoma deconvolution estimates with those derived from the widely used CNA-based analysis ichorCNA, an algorithm designed for low-coverage sequencing samples (15).

In approximately half of neuroblastoma cases, ichorCNA and methylation-based deconvolution yielded similar TF% estimates (Supplementary Table S10). Notable discrepancies highlighted the advantage of methylation-based deconvolution (Fig. 2B): In healthy controls, ichorCNA detected significant background noise TF% (6%–11%), whereas methylation-based deconvolution showed no sign of neuroblastoma [Fig. 2B (green)], demonstrating greater specificity. Two neuroblastoma cases in which methylation TF% differed significantly from the ichorCNA assessment were found to have genetic drivers favoring the methylation-based TF%. Sample cfNB4 [Fig. 2B (blue)] displayed a neuroblastoma deconvolution estimated 88% TF, in contrast to ichorCNA TF% of 14%. Based on cfDNA ONT sequencing data (8× coverage) of the known NRAS p.Gln61Lys mutation, a mutation-based TF% of 75% was calculated, closely aligning with methylation-based deconvolution scoring. Similarly, in the cfDNA sample cfNB9, ichorCNA predicted a 23% TF (despite prominent CNAs; see Supplementary Fig. S6), whereas methylation-based deconvolution estimated 56%. ddPCR confirmed an ALK p.Arg1275Gln mutation at a variant allele frequency (VAF) of 32.9%, yielding a mutation-based TF% of 65.8%, consistent with the methylation-based prediction. Reanalysis using an ONT-derived PoN (*n* = 12) and a PoN-free implementation (21) likewise revealed notable discrepancies in TF% estimates (>40%) compared with methylation-based deconvolution (Supplementary Table S11). In addition, deconvolution enables the estimation of contributions from other tissue types, providing further potential information. Taken together, these results demonstrate that methylation-based deconvolution is feasible in cfDNA and provides an accurate assessment of neuroblastoma contribution.

### meNBL markers for noninvasive monitoring of neuroblastoma disease progression

**Figure 3.**
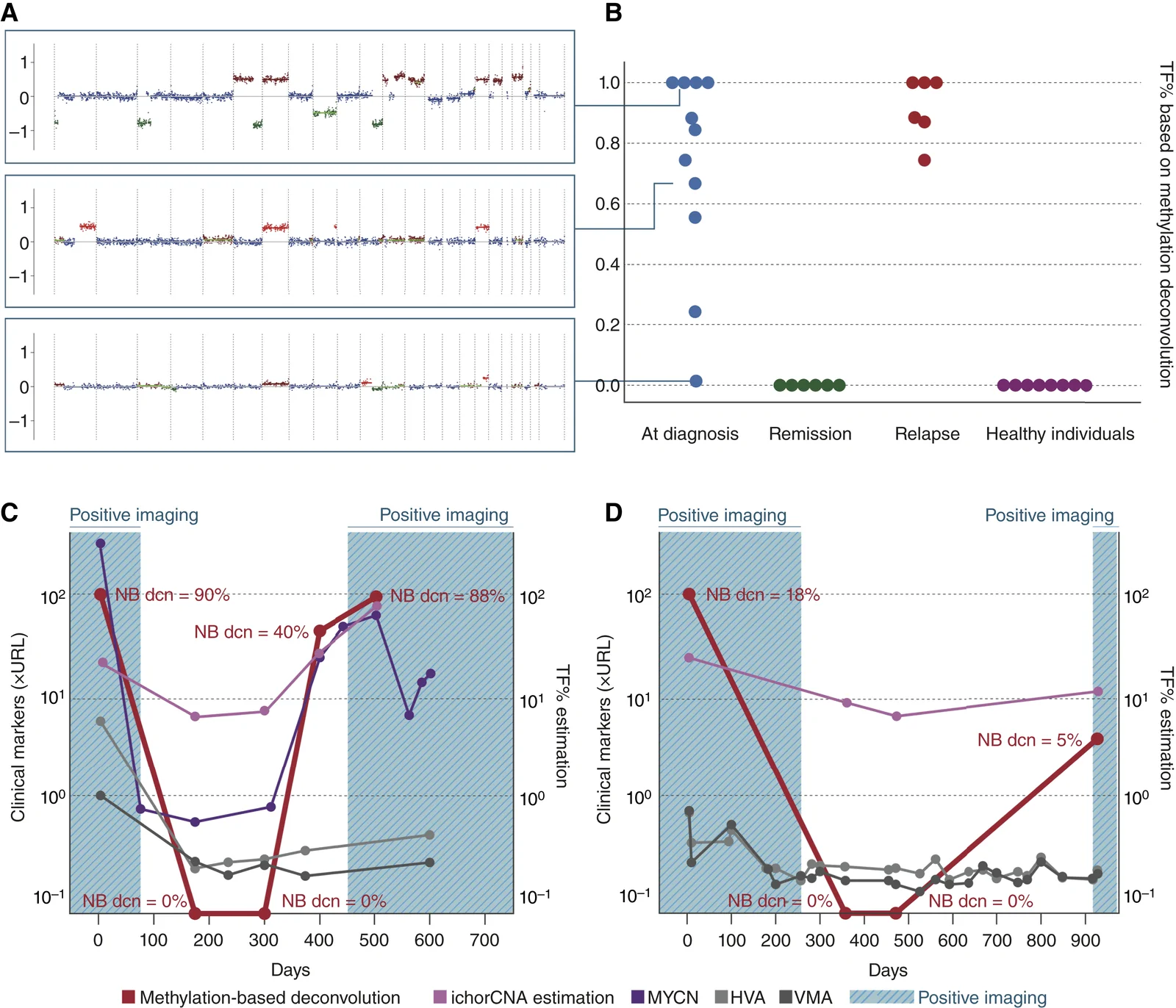
Neuroblastoma methylation markers enable disease monitoring and longitudinal follow-up. **A,** Representative CNV profiles derived from cfDNA samples collected at diagnosis from three patients with neuroblastoma with varying tumor fractions (TF%). From top to bottom, examples are shown for patients with high, intermediate, and low TF%. **B,** Tumor fraction (TF%) estimates obtained by methylation-based deconvolution using the 25 selected meNBL markers, shown for cfDNA samples collected at different disease stages: diagnosis (*n* = 11), remission (*n* = 6), relapse (*n* = 6), and healthy individuals (*n* = 8). Each dot represents an individual cfDNA sample, and the *y*-axis represents TF%. Arrows link representative TF% values in (**B**) to the corresponding CNV profiles shown in **A**. **C** and **D,** Longitudinal monitoring of neuroblastoma in two representative patients: a patient with MYCN amplification (**C**) and a patient without MYCN amplification (**D**). The *x*-axis denotes days from diagnosis. The left *y*-axis shows normalized values of established neuroblastoma disease markers, including MYCN copy number and urinary catecholamine metabolites [vanillylmandelic acid (VMA)/homovanillic acid (HVA)]. The right *y*-axis shows tumor fraction estimates derived from methylation-based deconvolution (NB dcn) using the neuroblastoma marker panel as well as ichorCNA. Blue shaded regions indicate time intervals during which gold-standard MIBG imaging confirmed active disease. In the MYCN-amplified case, methylation-based TF% tracking detected disease with sensitivity comparable with MYCN-based markers, whereas in the non-MYCN case, methylation-based detection mirrored imaging-based disease status.

Based on our findings that methylation-based liquid biopsy deconvolution using our meNBL DMRs detected neuroblastoma-derived cfDNA across all samples of high-risk patients with neuroblastoma at varying proportions while remaining undetectable (0%) in healthy donors (Fig. 3A; Supplementary Table S10), we hypothesized that the approach may address the need for noninvasive molecular monitoring in patients with neuroblastoma. To further validate neuroblastoma deconvolution accuracy, we applied the algorithm to cfDNA samples collected from patients with neuroblastoma during relapse and at molecularly confirmed remission time points (Supplementary Table S3). Methylation-based deconvolution analysis revealed the presence of neuroblastoma-specific cfDNA at diagnosis, its absence during confirmed remission time points, and marked elevation at relapse (Fig. 3B).

To directly validate this approach against the clinical standard of care for both neuroblastoma subtypes, those with trackable genetic aberrations and those without, we present a time course of serial samples from one patient with known *MYCN* amplification (Fig. 3C) and another with no detected neuroblastoma-associated genetic aberrations (Fig. 3D). In both patients, meNBL liquid biopsy predictions were concordant with clinical status determined by gold-standard meta-iodobenzylguanidine (MIBG) imaging, revealing detectable neuroblastoma-derived cfDNA during active disease and none during known remission. In the patient with *MYCN*-amplified disease, molecular assessment of disease presence was confirmed by ddPCR of cfDNA for *MYCN* amplification. In this patient, neuroblastoma-derived cfDNA was detectable with meNBL ∼100 days prior to clinically confirmed disease relapse and was consistent with plasma-based *MYCN* detection with ddPCR. These cases demonstrate that genomic molecular testing may outperform standard clinical assessment, including gold-standard urinary catecholamine biomarkers [vanillylmandelic acid (VMA)/homovanillic acid (HVA)]. Furthermore, due to its high accessibility via blood sampling, this liquid biopsy approach enables disease detection between imaging intervals, as demonstrated by longitudinal cfDNA profiling. Taken together, our results demonstrate that methylation-based deconvolution disease detection provides a robust and reliable estimator of neuroblastoma-derived cfDNA burden.

## Discussion

Serial disease assessment of pediatric solid tumors remains a significant challenge, hindering clinical decision-making and personalized treatment. Imaging-based techniques have inherent limitations, and children often require sedation, limiting their routine utility. In neuroblastoma, even disease-specific tracer-based imaging like MIBG brings its own challenges, as not all tumors exhibit MIBG uptake, and differentiated tumors without neuroblastic cells can still exhibit mature neuronal components with tracer uptake and positive imaging findings. Minimally invasive molecular monitoring can help address these problems, yet commercially available sequencing-based platforms fail to address this gap, as a significant proportion of high-risk neuroblastomas and pediatric solid tumors lack driver SNVs trackable in panel sequencing assays. There is a need for disease specific universal molecular markers that can be leveraged for broadly accessible and readily translatable liquid biopsy approaches.

In this study, we aimed to identify and leverage a robust neuroblastoma-specific methylation-based biomarker panel to develop a mutation-agnostic metric that can support the broad implementation of liquid biopsy-based disease assessment. Although this study represents a single-institution effort, the reproducibility of the methylation patterns and analytic performance observed here suggest broad applicability and warrant prospective validation in large, consortium-based cohorts. We utilized ONT sequencing, which offers key advantages over conventional methylation platforms like the EPIC 850K array, reduced representation bisulfite sequencing, or targeted capture panels. Unlike bisulfite-based methods requiring DNA conversion, ONT profiles native DNA methylation genome-wide while simultaneously capturing structural and sequence variants, critical for pediatric biopsies, in which tissue is often limited. In addition, our whole-genome methylation sequencing approach captures clusters of CpG sites, rather than isolated CpGs, which are less susceptible to technical artifacts and provide stronger potential biological context. ONT long-read capability also facilitates analysis of extended cfDNA fragments, an emerging biomarker in liquid biopsies. In addition to 5mC profiling, ctDNA ONT sequencing enables identification of 5-hmC modifications, which demonstrate prognostic significance in high-risk neuroblastoma ctDNA (22). Although no 5-hmC tissue atlas currently exists for deconvolution, this epigenetic mark remains an active area for further study. ONT whole-genome sequencing further supports fragmentomic analyses, which may further enhance sensitivity and biological discovery. Although ONT WGS currently remains more resource-intensive than targeted panel or low-pass WGS, it provides substantially richer multimodal information in a single assay, potentially reducing the need for parallel testing. In addition, real-time data acquisition offers opportunities for flexible sequencing duration and rapid analytic turnaround depending on clinical need. Emerging technologies, including Illumina’s five-base chemistry, further enable concurrent whole-genome and methylation profiling, underscoring the expanding technological landscape supporting integrated genomic and epigenomic liquid biopsy approaches.

Global methylation profiling distinguished high-risk neuroblastoma tumors from normal tissues, with PCA revealing three molecular subgroups consistent with prior reports (19). *MYCN*-amplified tumors clustered with an 11q-deleted case in the “*MYCN*-type” group, whereas *ATRX* and *SMARCA4* mutated tumors (both SWI/SNF complex components) formed a distinct chromatin-remodeling subgroup, and an *ALK*-mutated tumor segregated separately. The co-segregation of tumors with *MYCN* amplification and 11q deletion has been shown previously (19) and may stem from the inclusion of another patient with the rare co-occurrence of both *MYCN* amplification and 11q deletion. These genetic drivers, in addition to another included patient with the co-occurrence of *MYCN* amplification and *TERT* rearrangement, classically represent exclusive molecular subtypes; co-segregation of their genome-wide methylation patterns may offer insights into convergent molecular pathways. These subtype associations will require confirmation in larger independent cohorts and suggest the potential for complete molecular subtyping from a single diagnostic ONT run.

Integration of ONT-derived methylome maps with the normal human methylation atlas enabled the identification of 72 DMRs (meNBLs) relative to the atlas content. This atlas provides a high-resolution baseline of normal methylation signatures and may be considered a “complex” control reference, representing a comprehensive collection of cell type–specific biomarkers from healthy origins and enabling more accurate discrimination of neuroblastoma-specific methylation signals. As the atlas lacks fetal neural crest and its derivatives and neuroblastoma arises from neural crest–derived sympathoadrenal precursor cells, we performed the analysis both with and without neuronal cell types to ultimately encompass both neuronal-origin and broader pan-cancer markers. Notably, among the meNBLs shared between neuroblastoma and other advanced malignancies, we identified genes with reported methylation deregulation and biomarker utility, such as *SHOX2* (together with *RASSF1A*), *IRF6*, *TBX20*, *CCDC8*, and *DES*. Beyond their epigenetic deregulation, these genes participate in pathways with recurrent relevance to oncogenesis, including developmental and lineage programs, cytoskeletal remodeling, cellular plasticity, and tumor–stromal interactions (23). By integrating the selected 25 meNBLs into the existing human methylome atlas (13), we extended the reference to include neuroblastoma as a distinct cell type, enabling the development of a mutation-agnostic metric for estimating neuroblastoma-derived DNA in cfDNA through deconvolution. The concordance of these markers in the TARGET EPIC array data and an independent validation cohort reflects the robustness of our biomarker selection, and findings will need to be validated in larger cohorts.

This approach successfully identified heterogeneous ranges of neuroblastoma-derived cfDNA in patient samples, whereas no signal was detected in cfDNA from healthy donors or from high-risk patients with neuroblastoma in remission. The simultaneous assessment of 25 meNBLs was found to be more accurate than ichorCNA estimation of disease burden based on available somatic mutation data, and it exhibits no background noise, enabling the reliable detection of neuroblastoma-derived cfDNA even at very low levels. We note that copy number–based tumor fraction estimates using ichorCNA may vary depending on analytic parameters, particularly in the context of ONT-derived cfDNA data in which standardized PoNs are not yet established. However, in our analyses, key observations were consistent across multiple implementations.

In this context, other approaches such as the *RASSF1A* cfDNA methylation assay and cell-free RNA-based detection of TH and PHOX2B rely on single or dual markers, which may limit their sensitivity and specificity (9, 24). Moreover, *RASSF1A* has been reported as a pan-tumor marker, rather than being specific to neuroblastoma (9). Cell-free RNA-based assays also require specialized blood collection tubes that are not widely used in clinical practice, making them less practical to implement in routine diagnostic workflows compared with cfDNA-based methods. Furthermore, the methodology presented here for detecting meNBLs is readily adaptable for the identification of cancer-specific epigenetic markers in other malignancies. Beyond diagnosis, meNBLs may carry important mechanistic information not yet elucidated by genetic approaches, identifying novel DMRs with key roles in neurodevelopment, neuronal signaling, and tumor–microenvironment interactions. Hypermethylation of DMRs associated with neuronal maturation may reflect pathway dysregulation, and future work will explore the potential of the neuroblastoma methylome for drug target discovery.

In summary, we provide proof of concept that neuroblastoma-derived cfDNA methylation may provide a universal, mutation-agnostic metric for measuring disease burden across high-risk patients with neuroblastoma. Methylation-based cfDNA deconvolution provides accurate quantification of neuroblastoma cfDNA in the plasma, demonstrating the potential clinical utility for molecularly defined relapse detection. We plan to further validate these findings in a larger clinically annotated cohort of high-risk patients with neuroblastoma, ideally within clinical trials with matched imaging and surveillance data, to define the potential role of cfDNA methylation-based liquid biopsy testing in directing patient care.

## Supplementary Material

Supplementary Figure S1 Supplementary Figure S1: Identification of neuroblastoma-specific DMRs through comparative methylome analysis to normal human cell-type atlas. Total of 152 neuroblastoma DMRs were identified in comparison to all tissue types in the human reference atlas (13). Given that neuroblastoma arises from neural crest cells, the analysis was repeated with neuronal cell types excluded from the atlas resulting in the identification of additional 36 “neuronal” regions.

Supplementary Figure S2 Supplementary Figure S2: Gene Ontology (GO) enrichment analysis of 152 identified DMRs. The x-axis represents the gene ratio (the proportion of genes from the methylated gene list involved in each GO term), while the size of the dots corresponds to the number of genes associated with each process. The color gradient indicates the adjusted p-value (−log10 p.adjust), reflecting the statistical significance of enrichment.

Supplementary Figure S3 Supplementary Figure S3: DNA methylation analysis of neuroblastoma tumor samples reveals distinct methylation patterns. (A) Genome-wide DNA methylation levels were calculated in 7 neuroblastoma tumor gDNA and 3 PBL pools of 10 individuals each revealed globally reduced levels in neuroblastoma. Analysis is represented by violin plots with orange and white dots drawn at the distribution medians and means respectively. (B) Hierarchical clustering analysis based on methylation levels in neuroblastoma tumor and PBL pool samples reveal distinct molecular characteristics.

Supplementary Figure S4 Supplementary Figure S4: Identification of neuroblastoma-specific DMRs. Total of 72 neuroblastoma DMRs were identified in comparison to all tissue types in the human reference atlas. Average methylation values across each locus in the 7 primary neuroblastoma tissues as well as PBLs are shown.

Supplementary Figure S5 Supplementary Figure S5: Target distribution of 72 meNBLs across various genomic regions.

Supplementary Figure S6 Supplementary Figure S6: Comparison of circulating neuroblastoma-deconvolution with ichorCNA tumor fraction estimates in two neuroblastoma cfDNA samples showing discrepancies (cfNB4 and cfNB9).

Supplementary Table S1 Supplementary Table S1. Clinical and Genomic Characteristics of Neuroblastoma Tumor Samples Sequenced by ONT

Supplementary Table S2 Supplementary Table S2: Characteristics of cfDNA samples from neuroblastoma patients at diagnosis and controls analyzed by ONT sequencing

Supplementary Table S3 Supplementary Table S3: cfDNA samples from neuroblastoma patients at relapse and remission for deconvolution-based disease quantification

Supplementary Table S4 Supplementary Table S4: List of 60 neuroblastoma-associated genes from published studies analyzed for genomic aberrations in seven neuroblastoma tumors

Supplementary Table S5 Supplementary Table S5: Advanced tumor samples from O’Neill et al. (17) ONT cohort used for methylation comparison with neuroblastoma DMRs (n = 72)

Supplementary Table S6 Supplementary Table S6: List of 72 DMRs selected following comparative analysis with a normal human cell-type methylation atlas and cfDNA evaluation

Supplementary Table S7 Supplementary Table S7: Gene Ontology (GO) enrichment analysis performed on 152 neuroblastoma DMRs that were identified in comparison to all tissue types in the human reference atlas (including neurons) (13).

Supplementary Table S8 Supplementary Table S8: List of 188 neuroblastoma-specific DMRs identified by comparison with a normal human cell-type methylation atlas including and excluding neuronal tissues (13).

Supplementary Table S9 Supplementary Table S9: List of neuroblastoma-specific DMRs for cfDNA deconvolution analysis

Supplementary Table S10 Supplementary Table S10: Neuroblastoma cfDNA quantification in patients and controls: Deconvolution vs ichorCNA

Supplementary Table S11 Supplementary Table S11: Comparison of ichorCNA performance with provided vs generated panel of normal

## Data Availability

The data generated in this study are available within the article and its supplementary data files. The raw sequencing data generated in this study have been deposited in the NCBI Sequence Read Archive database and are accessible under the BioProject accession number PRJNA1433864. Any additional data including code used to generate the figures are available from the corresponding author upon request.
