# Supplementary material for: Epigenetic Liquid Biopsy Enables Universal Mutation-Agnostic Molecular Surveillance for High-Risk Neuroblastoma
Source: Clin Cancer Res. 2026 Jul 1;32(18):4084–94. doi: 10.1158/1078-0432.CCR-26-0457 (PMC13575560; doi:10.1158/1078-0432.CCR-26-0457)
Supplement: Supplementary file 1 [file ccr-26-0457_supplementary_figure_s1_suppfs1.docx]

**Supplementary Figure S1**: Identification of neuroblastoma-specific DMRs through comparative methylome analysis to normal human cell-type atlas. Total of 152 neuroblastoma DMRs were identified in comparison to all tissue types in the human reference atlas (13). Given that neuroblastoma arises from neural crest cells, the analysis was repeated with neuronal cell types excluded from the atlas resulting in the identification of additional 36 “neuronal” regions.
