# Supplementary material for: Epigenetic Liquid Biopsy Enables Universal Mutation-Agnostic Molecular Surveillance for High-Risk Neuroblastoma
Source: Clin Cancer Res. 2026 Jul 1;32(18):4084–94. doi: 10.1158/1078-0432.CCR-26-0457 (PMC13575560; doi:10.1158/1078-0432.CCR-26-0457)
Supplement: Supplementary file 2 [file ccr-26-0457_supplementary_figure_s2_suppfs2.docx]

**Supplementary Figure S2**: Gene Ontology (GO) enrichment analysis of 152 identified DMRs. The x-axis represents the gene ratio (the proportion of genes from the methylated gene list involved in each GO term), while the size of the dots corresponds to the number of genes associated with each process. The color gradient indicates the adjusted p-value (−log10 p.adjust), reflecting the statistical significance of enrichment.
