# Supplementary material for: Epigenetic Liquid Biopsy Enables Universal Mutation-Agnostic Molecular Surveillance for High-Risk Neuroblastoma
Source: Clin Cancer Res. 2026 Jul 1;32(18):4084–94. doi: 10.1158/1078-0432.CCR-26-0457 (PMC13575560; doi:10.1158/1078-0432.CCR-26-0457)
Supplement: Supplementary file 3 [file ccr-26-0457_supplementary_figure_s3_suppfs3.docx]

**A**

**B**

**Supplementary Figure S3**: DNA methylation analysis of neuroblastoma tumor samples reveals distinct methylation patterns. (A) Genome-wide DNA methylation levels were calculated in 7 neuroblastoma tumor gDNA and 3 PBL pools of 10 individuals each revealed globally reduced levels in neuroblastoma. Analysis is represented by violin plots with orange and white dots drawn at the distribution medians and means respectively. (B) Hierarchical clustering analysis based on methylation levels in neuroblastoma tumor and PBL pool samples reveal distinct molecular characteristics.
