# Supplementary material for: Epigenetic Liquid Biopsy Enables Universal Mutation-Agnostic Molecular Surveillance for High-Risk Neuroblastoma
Source: Clin Cancer Res. 2026 Jul 1;32(18):4084–94. doi: 10.1158/1078-0432.CCR-26-0457 (PMC13575560; doi:10.1158/1078-0432.CCR-26-0457)
Supplement: Supplementary file 4 [file ccr-26-0457_supplementary_figure_s4_suppfs4.docx]

**Supplementary Figure S4**: Identification of neuroblastoma-specific DMRs. Total of 72 neuroblastoma DMRs were identified in comparison to all tissue types in the human reference atlas. Average methylation values across each locus in the 7 primary neuroblastoma tissues as well as PBLs are shown.
