# Supplementary material for: Epigenetic Liquid Biopsy Enables Universal Mutation-Agnostic Molecular Surveillance for High-Risk Neuroblastoma
Source: Clin Cancer Res. 2026 Jul 1;32(18):4084–94. doi: 10.1158/1078-0432.CCR-26-0457 (PMC13575560; doi:10.1158/1078-0432.CCR-26-0457)
Supplement: Supplementary file 6 [file ccr-26-0457_supplementary_figure_s6_suppfs6.docx]

**Copy Number (log2 ratio)**

**Supplementary Figure S6**: Comparison of circulating neuroblastoma-deconvolution with ichorCNA tumor fraction estimates in two neuroblastoma cfDNA samples showing discrepancies (cfNB4 and cfNB9).
