# Supplementary material for: Epigenetic Liquid Biopsy Enables Universal Mutation-Agnostic Molecular Surveillance for High-Risk Neuroblastoma
Source: Clin Cancer Res. 2026 Jul 1;32(18):4084–94. doi: 10.1158/1078-0432.CCR-26-0457 (PMC13575560; doi:10.1158/1078-0432.CCR-26-0457)
Supplement: Supplementary file 7 [file ccr-26-0457_supplementary_table_s10_suppts10.docx]

**Supplementary Table S10:** Neuroblastoma cfDNA quantification in patients and controls: Deconvolution vs ichorCNA

|  | **Sample** | **Molecular classification** | **Neuroblastoma deconvolution %** | **ichorCNA TF %** | **Non-Tumor (Blood Cells) deconvolution %** | **Non-Tumor (Solid Tissues) deconvolution %** |
| --- | --- | --- | --- | --- | --- | --- |
| Matched to Tumor neuroblastoma cfDNA | cfNB1_106697 | non MYCN | 100 | 85.6 | 0 | 0 |
|  | cfNB2_115048 | non MYCN | 0.43 | 9.8 | 57.1 | 42.5 |
|  | cfNB3_113096 | ALK mutation | 73 | 54.2 | 8 | 18.9 |
|  | cfNB4_218072 | MYCN+TERT rearrangement | 87.9 | 14.0 | 0.0 | 12.1 |
|  | cfNB5_215323 | MYCN | 100 | 97.4 | 0.0 | 0.0 |
|  |  |  |  |  | 0 | 0 |
| Independent set neuroblastoma cfDNA | cfNB6_211373 | non MYCN | 23.1 | 61.7 | 72.2 | 4.7 |
|  | cfNB7_210823 | non MYCN | 100 | 55.7 | 0.0 | 0.0 |
|  | cfNB9_218011 | ALK mutation | 55.7 | 22.8 | 6.8 | 37.5 |
|  | cfNB10_214232 | MYCN | 66.7 | 13.9 | 0.0 | 33.3 |
|  | cfNB11_216963 | MYCN+ALK amp | 73 | 59.9 | 5.0 | 21.5 |
|  | cfNB12_202104 | MYCN+ALK mutation | 100 | 58.0 | 0.0 | 0.0 |
|  | cfNB13_107298 | MYCN | 90 | 29.6 | 0.0 | 9.6 |
|  |  |  |  |  | 0 | 0 |
| Adult ctrl cfDNA | cfCtrl_Ad1 | NA | 0 | 6.35 | 39.00 | 61.00 |
|  | cfCtrl_Ad2 | NA | 0 | 6.27 | 48.30 | 51.70 |
|  | cfCtrl_Ad3 | NA | 0 | 9.9 | 70.2 | 29.8 |
|  | cfCtrl_Ad4 | NA | 0 | 10.88 | 47.8 | 52.2 |
|  | cfCtrl_Ad6 | NA | 0 | 8.52 | 50.30 | 49.70 |
|  |  |  |  |  | 0 | 0 |
| Pediatric Ctrl cfDNA | cfCtrlPd2 | NA | 0 | NA | 34.7 | 65.3 |
|  | cfCtrlPd3 | NA | 0 | 10.08 | 83.4 | 16.6 |
|  | cfCtrlPd5 | NA | 0 | 11.25 | 50 | 50 |
