# Supplementary material for: Epigenetic Liquid Biopsy Enables Universal Mutation-Agnostic Molecular Surveillance for High-Risk Neuroblastoma
Source: Clin Cancer Res. 2026 Jul 1;32(18):4084–94. doi: 10.1158/1078-0432.CCR-26-0457 (PMC13575560; doi:10.1158/1078-0432.CCR-26-0457)
Supplement: Supplementary file 8 [file ccr-26-0457_supplementary_table_s11_suppts11.docx]

**Supplementary Table S11:** Comparison of ichorCNA performance with provided vs generated panel of normal

|  | **Default PoN** | **PoN 12 controls** | | | **No PoN** | | |
| --- | --- | --- | --- | --- | --- | --- | --- |
| **sample name** | **TF%** | **TF%** | **Delta from default** | **ttest** | **TF%** | **Delta from default** | **ttest** |
| cfNB2 | 0.09818 | 0.1226 | -0.02442 | 0.301438 | 0.1198 | -0.02162 | 0.157586 |
| cfNB4 | 0.1397 | 0.1201 | 0.0196 |  | 0.1289 | 0.0108 |  |
| cfNB5 | 0.9735 | 0.9413 | 0.0322 |  | 0.932 | 0.0415 |  |
| cfNB12 | 0.5797 | 0.5839 | -0.0042 |  | 0.4502 | 0.1295 |  |
| cfNB3 | 0.542 | 0.55 | -0.008 |  | 0.5428 | -0.0008 |  |
| cfNB9 | 0.2284 | 0.2296 | -0.0012 |  | 0.6587 | -0.4303 |  |
| cfNB10 | 0.1389 | 0.1369 | 0.002 |  | 0.1444 | -0.0055 |  |
| cfNB1 | 0.8556 | 0.8625 | -0.0069 |  | 0.8553 | 0.0003 |  |
| cfNB7 | 0.5572 | 0.5805 | -0.0233 |  | 0.5721 | -0.0149 |  |
| cfNB6 | 0.617 | 0.635 | -0.018 |  | 0.6214 | -0.0044 |  |
| cfNB13 | 0.2962 | 0.2891 | 0.0071 |  | 0.7719 | -0.4757 |  |
| cfNB11 | 0.5994 | 0.8391 | -0.2397 |  | 0.8308 | -0.2314 |  |
| **Avg Delta from defualt** |  |  | -0.02207 |  |  | -0.08354 |  |

*Tumor fraction (TF%) in cfDNA samples was estimated using ichorCNA with three different panels of normals (PoN): the original PoN from the ichorCNA data repository (default PoN in the table above), a PoN generated from 12 independent healthy control samples (PoN 12 controls in the table above), and no PoN. Delta values represent the difference between TF% estimated using the original PoN and the respective custom PoN. Overall, the distribution of TF% estimates was not statistically significant whether generated by the original PoN versus either custom PoN.*
