# Supplementary material for: Epigenetic Liquid Biopsy Enables Universal Mutation-Agnostic Molecular Surveillance for High-Risk Neuroblastoma
Source: Clin Cancer Res. 2026 Jul 1;32(18):4084–94. doi: 10.1158/1078-0432.CCR-26-0457 (PMC13575560; doi:10.1158/1078-0432.CCR-26-0457)
Supplement: Supplementary file 9 [file ccr-26-0457_supplementary_table_s1_suppts1.docx]

**Supplementary Table S1.** Clinical and Genomic Characteristics of Neuroblastoma Tumor Samples Sequenced by ONT

* TERT: Chr5:1291626 INS and Chr5:1273682-1273791 DEL

** TERT: Chr5:1273349-1273501 DEL
