# Supplementary material for: Epigenetic Liquid Biopsy Enables Universal Mutation-Agnostic Molecular Surveillance for High-Risk Neuroblastoma
Source: Clin Cancer Res. 2026 Jul 1;32(18):4084–94. doi: 10.1158/1078-0432.CCR-26-0457 (PMC13575560; doi:10.1158/1078-0432.CCR-26-0457)
Supplement: Supplementary file 12 [file ccr-26-0457_supplementary_table_s4_suppts4.docx]

**Supplementary Table S4:** List of 60 neuroblastoma-associated genes from published studies analyzed for genomic aberrations in seven neuroblastoma tumors
