# Supplementary material for: Epigenetic Liquid Biopsy Enables Universal Mutation-Agnostic Molecular Surveillance for High-Risk Neuroblastoma
Source: Clin Cancer Res. 2026 Jul 1;32(18):4084–94. doi: 10.1158/1078-0432.CCR-26-0457 (PMC13575560; doi:10.1158/1078-0432.CCR-26-0457)
Supplement: Supplementary file 14 [file ccr-26-0457_supplementary_table_s9_suppts9.docx]

**Supplementary Table S9:** List of neuroblastoma-specific DMRs for cfDNA deconvolution analysis

2
